# Developing a Machine Learning System for Identification of Severe Hand, Foot, and Mouth Disease from Electronic Medical Record Data

**DOI:** 10.1038/s41598-017-16521-z

**Published:** 2017-11-27

**Authors:** Guangjian Liu, Yi Xu, Xinming Wang, Xutian Zhuang, Huiying Liang, Yun Xi, Fangqin Lin, Liyan Pan, Taishan Zeng, Huixian Li, Xiaojun Cao, Gansen Zhao, Huimin Xia

**Affiliations:** 1 Institute of Pediatrics, Guangzhou Women and Children’s Medical Center, Guangzhou Medical University, Guangzhou, China; 2 Department of Infectious Diseases, Guangzhou Women and Children’s Medical Center, Guangzhou Medical University, Guangzhou, China; 3 School of Computer, South China Normal University, Guangzhou, China; 4 School of Mathematical Sciences, South China Normal University, Guangzhou, China; 5 Department of Research, Education and Data Management, Guangzhou Women and Children’s Medical Center, Guangzhou Medical University, Guangzhou, China; 6 Department of Pediatric Surgery, Guangzhou Women and Children’s Medical Center, Guangzhou Medical University, Guangzhou, China

## Abstract

Children of severe hand, foot, and mouth disease (HFMD) often present with same clinical features as those of mild HFMD during the early stage, yet later deteriorate rapidly with a fulminant disease course. Our goal was to: (1) develop a machine learning system to automatically identify cases with high risk of severe HFMD at the time of admission; (2) compare the effectiveness of the new system with the existing risk scoring system. Data on 2,532 HFMD children admitted between March 2012 and July 2015, were collected retrospectively from a medical center in China. By applying a holdout strategy and a 10-fold cross validation method, we developed four models with the random forest algorithm using different variable sets. The prediction system HFMD-RF based on the model of 16 variables from both the structured and unstructured data, achieved 0.824 sensitivity, 0.931 specificity, 0.916 accuracy, and 0.916 area under the curve in the independent test set. Most remarkably, HFMD-RF offers significant gains with respect to the commonly used pediatric critical illness score in clinical practice. As all the selected risk factors can be easily obtained, HFMD-RF might prove to be useful for reductions in mortality and complications of severe HFMD.

## Introduction

Hand, foot, and mouth disease (HFMD) is a common childhood illness caused by a group of enteroviruses such as enterovirus 71 (EV71) and coxsackievirus A16 (CA16)^1,2^. In recent years, outbreaks of HFMD have increased, and more and more severe cases and fatalities have appeared throughout most of the Asia-Pacific Region countries^3^. For example, data reported by the Western Pacific Regional Office of the World Health Organization showed that in China, a total of 2,468,174 cases of HFMD including 220 deaths were reported in 2016^4^, representing a substantial public health threat.

HFMD is characterized by fever, general malaise, sore throat and vesicular eruptions on the hands, feet, tongue and oral mucosa^5^. Severe cases can also involve serious neurological, respiratory or circulatory complications, such as meningitis, encephalitis, cardiorespiratory failure, acute flaccid paralysis or even death^6–8^. Although the prognoses in most cases are good, children with severe HFMD usually have no typical clinical manifestations in the early stage and rapidly progress to severe or fatal disease in a short term. Consequently, the identification of high-risk patients who are going to develop a severe form of HFMD is a key goal in the management of this disease.

A variety of predictive factors have been utilized by clinicians and researchers to identify cases whose conditions are likely to deteriorate. A meta-analysis of 19 observational studies has identified variables that conferred risk for rapid progression to severe disease, including fever duration, peak temperature, age, vomiting, neutrophil count, hyperglycemia and EV71 infection^9^. Other groups have sought to correlate clinically useful biomarkers such as cytokines with disease severity^10–12^. Several single nucleotide polymorphisms might correlate with both susceptibility and progression of severe disease^13,14^. Moreover, MRI-related variables have also been involved in the identification of severe HFMD children with state-of-the-art machine learning algorithms^15^.

The most commonly used scoring system for disease severity in pediatric intensive care units is the pediatric critical illness score (PCIS), which was drawn up by the emergency group of Chinese pediatric society, Chinese medical association in 1995. One of the greatest challenges with PCIS application in HFMD lies in that it is not designed specifically for HFMD and thus cannot adequately evaluate the severity of HFMD^16^. To our knowledge, no simple, real-time and automatic prediction tools for severe HFMD identification have emerged as clinically practicable since 1995. Therefore, the interest of this study consists in staging HFMD early in hospitalization to provide effective and timely medical intervention.

The widespread implementation of Electronic Medical Record (EMR) brings the promise of abundant data resources for research purposes such as better prediction of clinical deterioration^17^. A number of machine learning algorithms and models have shown their advantages in improving real-time identification of sepsis shock, heart failure and other diseases^18–22^. In this paper we show whether machine learning methods and clinical data obtained from a relatively large population of HFMD patients can be efficiently applied to address the severe HFMD identification problems. We identified relevant predictive variables in severe HFMD and compared models of different complexity to provide to physicians a simple, robust and automatic decision-making support system. We also provided a comparison between the system and PCIS currently used in clinic.

## Results

### Basic Characteristics

Table 1 shows the baseline characteristics of all subjects. A total of 2,532 HFMD hospitalizations were included in this study (mean age, 24.11 [standard derivation 17.14] months; 1,715 males [67.73%]). Of these hospitalizations, 365 (14.42%) progressed to the severe stage with complications including encephalitis (n = 237), meningitis (n = 36), acute flaccid paralysis (n = 19), cardiorespiratory failure (n = 258), or death (n = 5). For the population of severe cases, the median time from illness onset to diagnosis was 2 days (interquartile range 1–3), and 1 day (interquartile range 0–2) for diagnosis to severity. Distributions of the onset-to-diagnosis and diagnosis-to-severity time interval showed that 79.18% (289/365) of cases sought treatment in hospital within 3 days of illness onset and 74.52% (272/365) of cases developed severe complications after 1 day of admission (Supplementary Figure S1).

**Table 1:** Baseline Characteristics.

Characteristics	Total Data Set (n = 2532)	Training Set (n = 1899)	Test Set (n = 633)	χ^2^/t	P-value
Stage (Severe/Mild)	365/2167	274/1625	91/542	0.13	0.732
Gender (Male/Female)	1715/817	1282/617	433/200	0.17	0.677
Vomiting (Yes/No)	393/2139	292/1607	101/532	0.12	0.727
Age (month)	24.11 ± 17.14	24.19 ± 16.94	23.86 ± 17.72	0.43	0.669
Respiratory rate (/min)	27.27 ± 5.61	27.27 ± 6.16	27.27 ± 3.46	−0.01	0.989
Peak temperature (°C)	39.08 ± 0.71	39.08 ± 0.71	39.09 ± 0.72	−0.30	0.763
Fever duration (day)	2.50 ± 1.76	2.52 ± 1.79	2.47 ± 1.67	0.65	0.513
Blood glucose (mmol/L)	5.56 ± 6.14	5.54 ± 1.35	5.62 ± 1.42	−1.21	0.226
Platelet (10^9^/L)	322.03 ± 93.06	321.61 ± 92.70	323.29 ± 94.20	−0.27	0.790
Percentage of lymphocytes (%)	39.35 ± 16.41	39.53 ± 16.50	38.82 ± 16.16	0.95	0.345
Lactate dehydrogenase (U/L)	310 (73)	309 (72)	313 (73)	0.50	0.620^*^
Alkaline phosphatase (IU/L)	178.96 ± 66.34	179.12 ± 61.69	178.46 ± 78.73	0.49	0.624
Creatine kinase (IU/L)	114 (80)	114 (80)	113 (80)	0.30	0.761^*^
Creatine kinase-MB (IU/L)	32.34 ± 18.07	32.31 ± 17.98	32.42 ± 18.36	−0.12	0.904
Creatinine (µmol/L)	24.21 ± 5.84	24.13 ± 5.73	24.45 ± 6.15	−1.38	0.169
Uric acid (µmol/L)	285.41 ± 83.18	285.33 ± 83.26	285.66 ± 83.00	−0.09	0.932
Blood chlorine (mmol/L)	100.31 ± 2.34	100.31 ± 2.25	100.29 ± 2.61	0.22	0.830
Alanine aminotransferase (IU/L)	18 (11)	18 (11)	18 (11)	−0.75	0.456^*^

Data are mean ± standard derivation or median (interquartile range). The Chi-square test was used for comparison of categorical variables and the two-sample t test for continuous variables between the training set and the test set.

^*^Transformed logarithmically to assume a near-normal distribution for t-test.

All the hospitalizations were randomly sorted 7:3 into a training set with 1,899 patients and a test set with 633 patients. There is no significant difference between the training and test sets in terms of baseline variables (Table 1).

### Feature Selection

**Figure 1:**
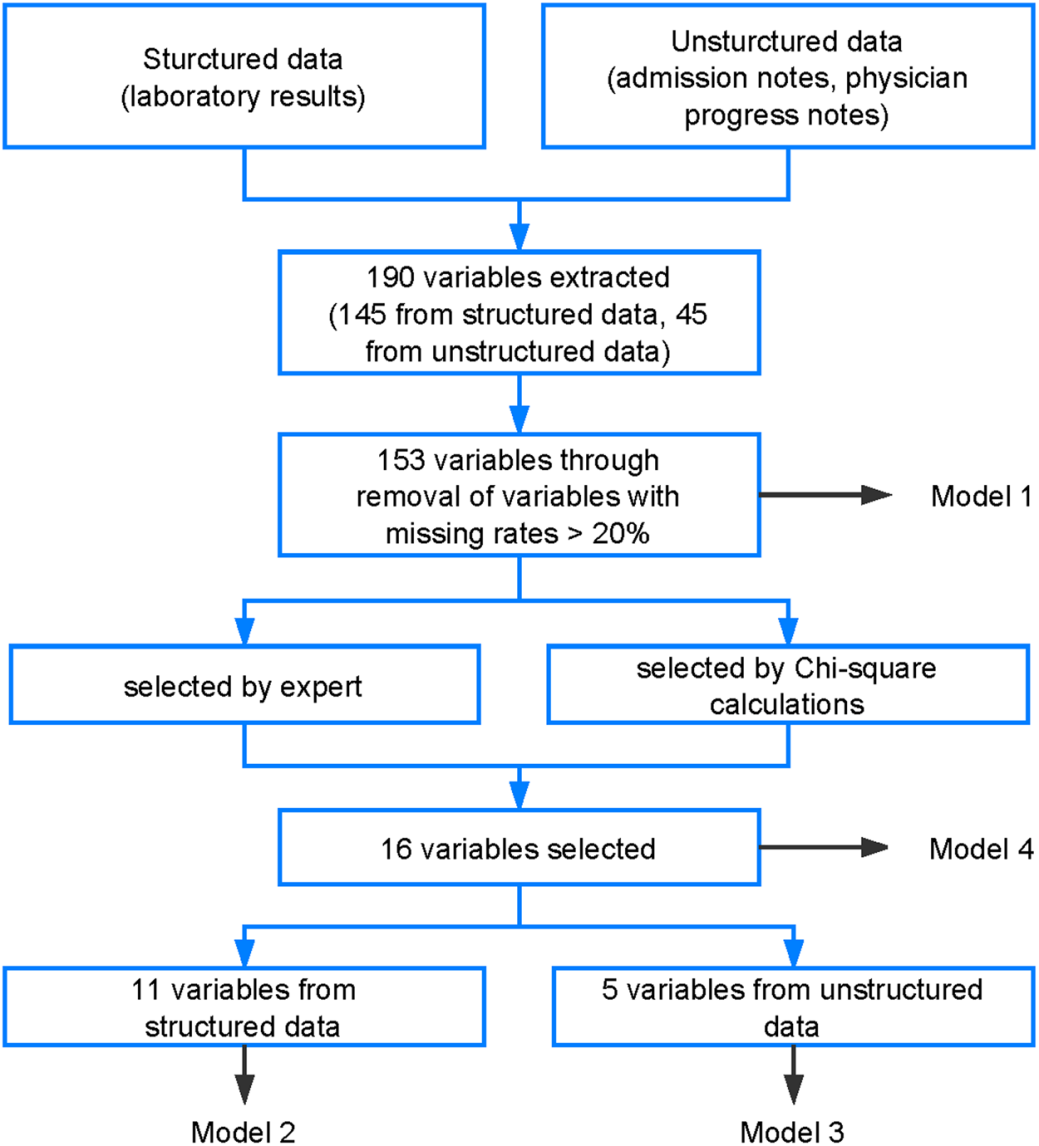
Identification and selection procedure of clinical features for machine learning models.

After removal of variables with missing rate >20%, a total of 153 variables consisting of laboratory results, vital signs, symptoms and demographics were extracted from the structured and unstructured data of EMR (Fig. 1). Then, we selected the predictors related to severe HFMD according to literature review, expert clinician opinion and univariate analysis. Eleven variables were chosen from the structured laboratory results: blood glucose, platelet, percentage of lymphocytes, lactate dehydrogenase, alkaline phosphatase, creatine kinase, creatine kinase-MB, creatinine, uric acid, blood chlorine, alanine aminotransferase, and five from the unstructured free text: age, respiratory rate, peak temperature, fever duration, vomiting.

Predictive efficiencies of the selected variables were supported by the Chi-square test or the two sample t-test to clarify the differences between the severe and mild HFMD groups. As expected, the null hypothesis was rejected for all the variables (Table 2), which showed that their statistical distributions should be considered to be different and the differences to be significant. These results confirmed the importance of variables from both the structured and unstructured data in identifying severe HFMD.

**Table 2:** Characteristics of severe and mild HFMD groups.

Variables	Severe HFMD	Mild HFMD	χ^2^/t	p-value
Vomiting (Yes/No)	133/365	260/2167	142.31	<0.001
Age (month)	30.58 ± 20.01	23.02 ± 16.36	6.85	<0.001
Respiratory rate (/min)	28.72 ± 12.94	27.03 ± 2.87	2.49	0.013
Peak temperature (°C)	39.21 ± 0.57	39.06 ± 0.73	4.40	<0.001
Fever duration (day)	3.27 ± 1.93	2.38 ± 1.69	8.30	<0.001
Blood glucose (mmol/L)	6.14 ± 1.39	5.47 ± 1.34	8.78	<0.001
Platelet (10^9^/L)	353.53 ± 108.05	316.72 ± 89.23	6.07	<0.001
Percentage of lymphocytes (%)	39.77 ± 16.43	36.90 ± 16.15	−3.09	0.002
Lactate dehydrogenase (U/L)	320 (67)	228 (250)	−13.10	<0.001^*^
Alkaline phosphatase (IU/L)	163.19 ± 43.48	181.61 ± 69.12	−6.69	<0.001
Creatine kinase (IU/L)	120 (53)	113 (79)	−3.94	<0.001^*^
Creatine kinase-MB (IU/L)	34.56 ± 17.84	19.12 ± 13.13	−17.82	<0.001
Creatinine (µmol/L)	24.44 ± 6.93	23.60 ± 5.94	−2.43	0.015
Uric acid (µmol/L)	287.51 ± 82.58	272.98 ± 85.74	−3.09	0.002
Blood chlorine (mmol/L)	100.00 ± 3.46	100.36 ± 2.09	2.74	0.006
Alanine aminotransferase (IU/L)	19 (6)	18 (11)	−2.58	0.010^*^

Data are mean ± standard derivation or median (interquartile range). The Chi-square test was used for comparison of categorical variables and the two-sample t test for continuous variables between the severe group and the mild group.

^*^Transformed logarithmically to assume a near-normal distribution for t-test.

### Prediction Results Using Machine Learning Algorithms

**Figure 2:**
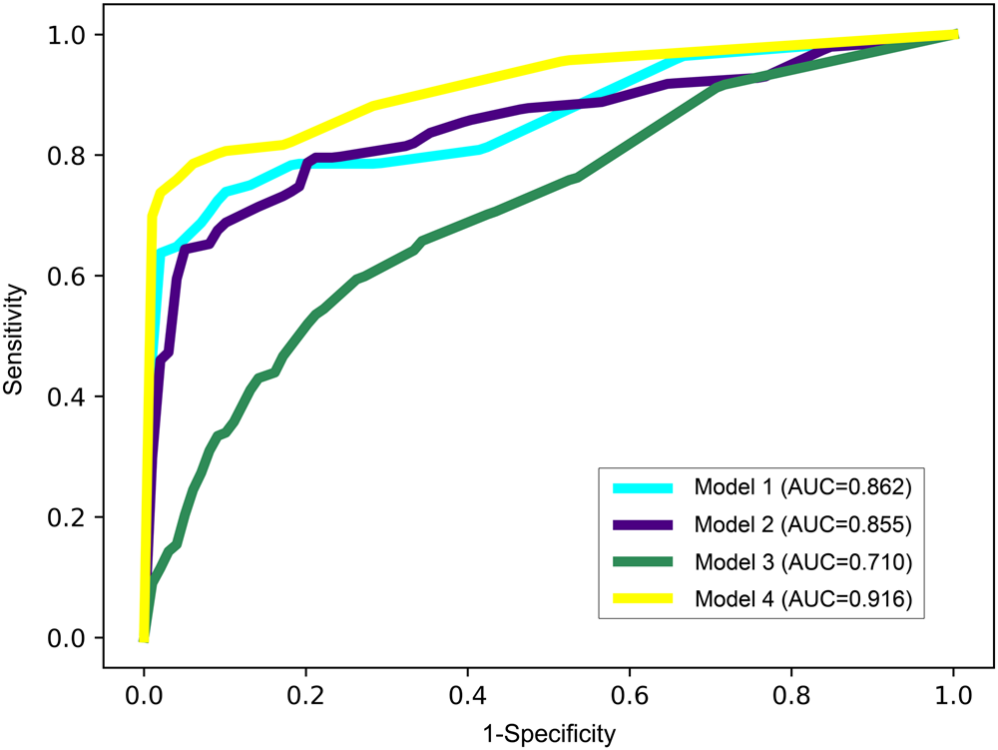
The receiver operating characteristic (ROC) curves of the four random forest models.

Figure 2 showed the receiver operating characteristic (ROC) curves for the four RF models basing on different variable sets. With all the 153 variables, the first model (Model 1) identified severe HFMD with a reasonable area under the ROC curve (AUC) of 0.862. Using the structured variables alone, the second model (Model 2) had comparable performance to that of Model 1, with an AUC of 0.855. Yet using the unstructured variables alone, the performance of the third model (Model 3) declined seriously to an AUC of 0.710. Interestingly, if both kinds of variables were used, the fourth model (Model 4) had even greater predictive ability than Model 1, yielding an AUC of 0.916. Overall, the performance of only one kind of variables lagged behind their combined performance in a RF model, demonstrating the importance of defining complex predictors and of combining their efficiencies in nonlinear models. This conclusion was supported by the similar changing patterns of AUC when applying other machine learning algorithms such as support vector machine, XGBoost, logistic regression and multi-layer perceptrons, although these algorithms performed a little worse than or equally to RF (Supplementary Figure S2).

**Figure 3:**
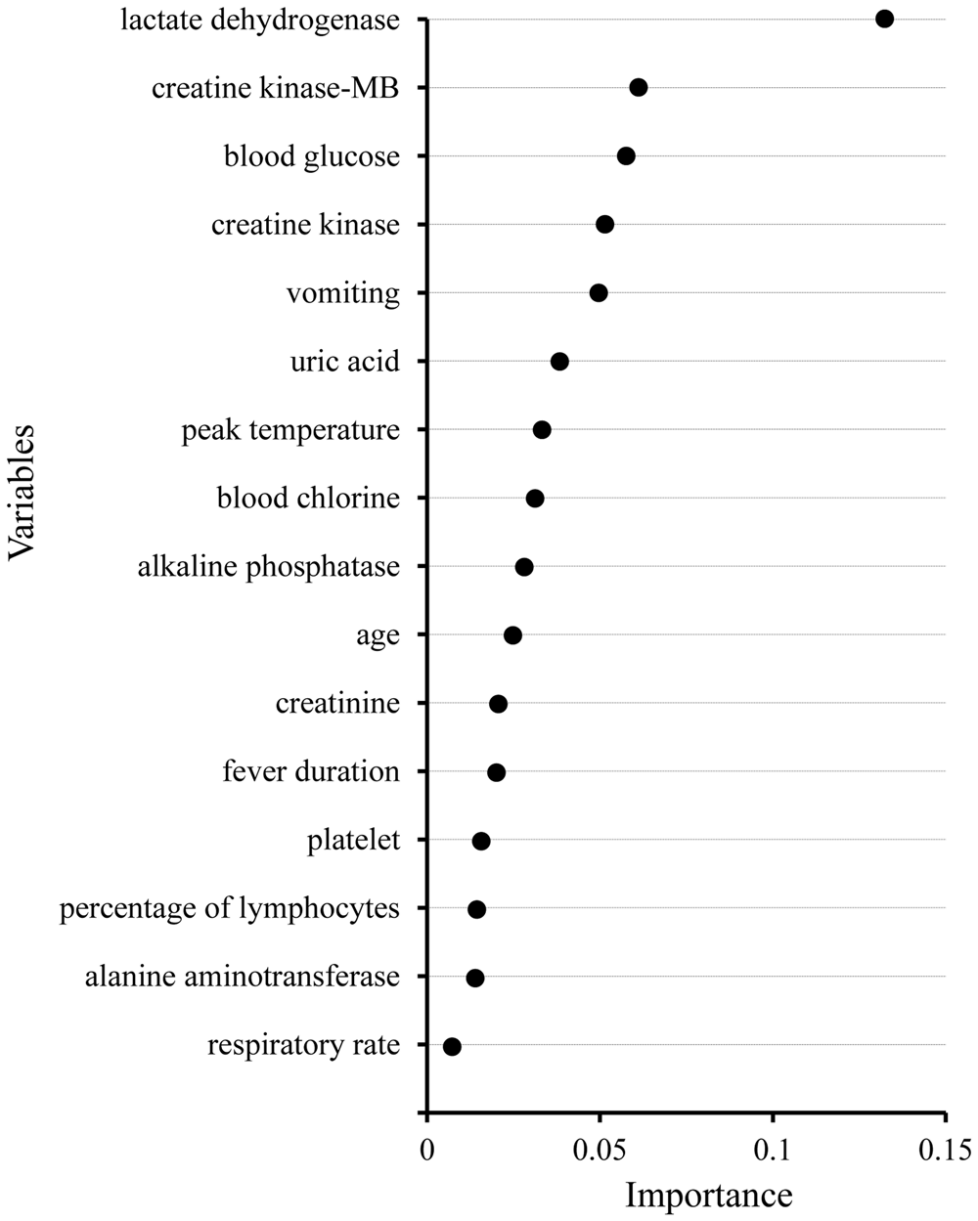
The importance of the 16 variables of the fourth random forest model.

We computed the importance score for each variable to identify the important features used by the RF classifier. The larger the score is, the more important the variable is. As shown in Fig. 3, the most important predictive variable was lactate dehydrogenase (LDH), followed by creatine kinase-MB (CK-MB), blood glucose and creatine kinase (CK). The symptom vomiting was the fifth important factor. Only a very small importance was found in respiratory rate, alanine aminotransferase, percentage of lymphocytes and platelet.

### Overall Comparison of Predictive systems

**Figure 4:**
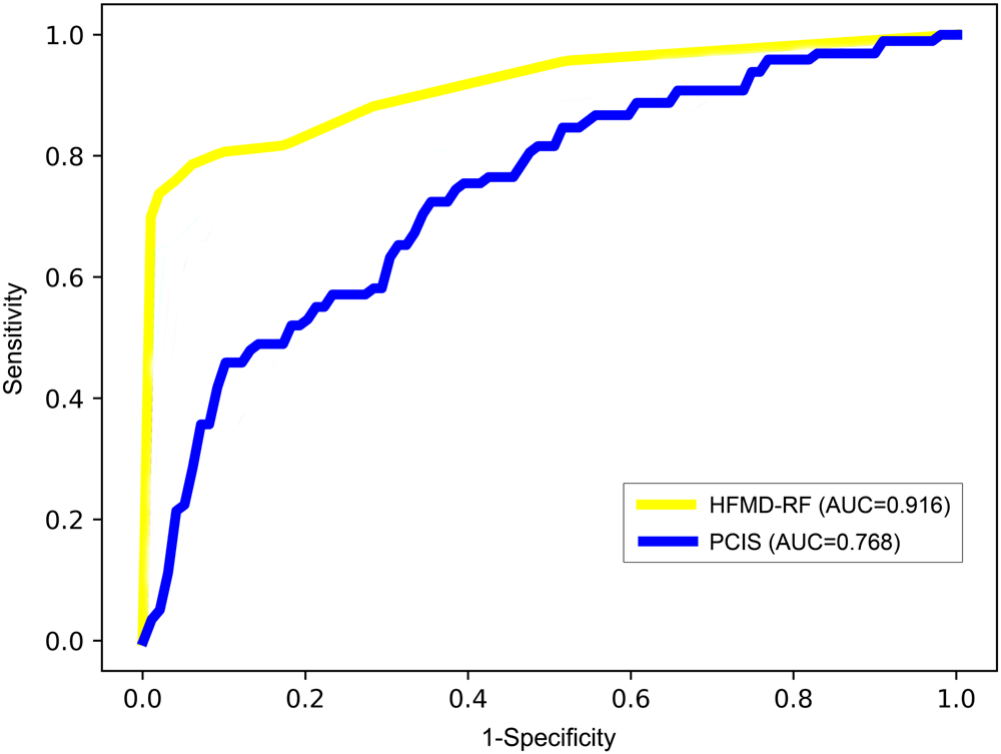
The receiver operating characteristic (ROC) curves of the HFMD-RF system and the pediatric critical illness score (PCIS).

Owing to its superb performance, we built a prediction system based on Model 4 of RF for predicting the progression of HFMD and named it HFMD-RF. The HFMD-RF system was then compared with the clinically used scoring system, PCIS. ROC curves for these two systems are plotted in Fig. 4. Their performance was presented in Table 3. In comparison to PCIS in the training and test sets, our system’s AUC, sensitivity, specificity and accuracy were significantly improved. The p-values comparing the two systems are less than 0.001 except for the sensitivity in the test set (p = 0.017). Our system’s sensitivity and specificity numerically improved by 21.3% and 25.2% in the test set. The accuracy and AUC of our model were both 0.916, which increased by 24.7% and 18.8% in comparison with PCIS.

**Table 3:** Performance comparison of HFMD-RF against the pediatric critical illness score (PCIS).

		Sensitivity	Specificity	Accuracy	AUC
Performance					
Training Set	PCIS	0.631	0.742	0.726	0.723
	HFMD-RF	0.807	0.969	0.945	0.919
Test Set	PCIS	0.670	0.753	0.741	0.768
	HFMD-RF	0.824	0.931	0.916	0.916
		**Sensitivity (improvement, P value)**	**Specificity (improvement, P value)**	**Accuracy (improvement, P value)**	**AUC (improvement, P value)**
Performance comparison					
Training Set	HFMD-RF against PCIS	27.9%, P < 0.001	30.6%, P < 0.001	30.2%, P < 0.001	26.8%, P < 0.001
Test Set	HFMD-RF against PCIS	21.3%, P = 0.017	25.2%, P < 0.001	24.7%, P < 0.001	18.8%, P < 0.001

The Chi-square test was used for comparison of Sensitivity, Specificity and Accuracy, and the two-sample t test for AUC between HFMD-RF and PCIS.

## Discussion

Due to the rapid progression of symptoms and the ambiguous boundary between disease stages, patients with severe HFMD are at high risk of insufficient quality of care and poor post discharge outcomes^8^. Rapid identification of patients with severe HFMD is thus urgently required to prevent deterioration and to reduce acute mortality. In this study, we developed and validated a prediction system for progression to severe HFMD through automated analysis of EMR data. Our major findings are as follows: (1) The prediction system can provide accurate identification of severe HFMD with an AUC of 0.916, basing on 16 clinical variables collected at the time of admission. (2) Results have shown that LDH, CK-MB, blood glucose, CK and vomiting are the top five indicators associated with the risk of severe HFMD. (3) Our system achieved significantly higher performance than application of the existing PCIS in clinic.

Special expertise and resource may be required to implement a sophisticated machine learning system in an EMR. As a result, the tradeoff between cost of implementation and benefit of performance improvement with sophisticated approaches should be considered. Through a process using literature review, expert clinician opinion, univariate analysis and machine learning, we built and compared four RF models based on different variable sets. Model 1 of 153 variables obtained a moderate performance in terms of AUC, a measurement of global classification. Model 2 was superior to Model 1 because it got a comparable AUC using only 11 structured variables and was relatively easier to implement. Model 3 and 4 are likely more difficult to implement because of their dependence on processing of unstructured data. However, this cost may be worth the improved performance in Model 4, which obtained the best AUC by integrating five unstructured and eleven structured variables. Combining the analysis of free-text notes and structured laboratory results appears to have the best predictive performance.

A similar machine learning model has been built recently for risk prediction of severe HFMD and achieved an AUC of up to 0.985 with several clinical and MRI-related features^15^, representing a great advance. However, MRI examination requires special equipment and is time- and cost-consuming, making this model difficult in general medical practice or for every patient. Jun Qiu *et al*. developed another model with good discrimination (AUC > 0.9) using only four laboratory parameters^23^. Whereas this model has a different purpose and can only discriminate children with high mortality risk from severe HFMD cases. Therefore, the present identification system HFMD-RF was developed to provide accurate risk stratification for mild HFMD patients in a timely and effective manner with easily-obtained variables.

Comparing HFMD-RF with the existing scoring system PCIS, HFMD-RF demonstrated superior performance in both the training and test sets in terms of sensitivity, specificity, accuracy and AUC. These results reveal that HFMD-RF may be more clinically practicable for the identification of severe HFMD patients than PCIS. One reason for this finding may lie in that we used 16 variables selected specifically for HFMD while the 10 variables in PCIS (see Methods) are aimed at all the serious diseases in pediatric intensive care units. Besides, unlike PCIS which combines sub-score in each variable linearly to create an overall score^24,25^, we used RF so that distinct variables can be integrated in nonlinear models for optimal prediction^26^.

Our research indicates that myocardial enzymes LDH, CK-MB, CK are very important risk factors for severe HFMD, as have been found in previous studies^27–29^. LDH is a cytoplasmic enzyme and its release into plasma reflects the degree of tissue damage. CK-MB and CK, which are mostly found in myocardial cells, are well-known and sensitive indicators of myocardial injury. Compared with the mild HFMD group, the severe HFMD group had significantly higher LDH, CK-MB, CK levels, indicating the degree of myocardial injury differs in children with HFMD. This suggests that LDH, CK-MB and CK may be valuable diagnostic markers in predicting the disease severity in HFMD children before they develop pulmonary oedema, pulmonary haemorrhage or heart failure.

The selected attributes in HFMD-RF can also be used to provide simple biomedical discriminatory rules for severe HFMD identification. We provide means or medians for the mild and severe HFMD groups, which can be of help in physicians’ decision-making process. Most of the variables used in our system have been reported previously, such as age, peak temperature, fever duration, vomiting, blood glucose and so on^1,9,30,31^. Besides, our results also indicated the prediction significance of other variables including alkaline phosphatase, creatinine and uric acid that are mainly related with the status of the liver and kidney and are not currently used as predictive factors in this disease. These newly found predictors might be useful to understand the potential cause of severe HFMD from a medical point of view.

It is crucially important to note that different combination lists of variables might exist with similar predictive performance due to the uncertainty space of the solutions in decision-making problems^32^. Besides superior performance, the variable list used in our model has a great advantage in that each variable was present in the EMR for clinical care. No additional reporting structure or extra clinical assessments were required.

Given these benefits, we plan to deploy the HFMD-RF system at our center to facilitate interventions that targeted hospitalized HFMD children. To implement this, EMR data will be exported to a secure server on which the system will run; the identification result of the system will be put back into the EMR for care delivery. In this way, our model can be replicated easily in other hospitals without considering EMR vendor.

Some important limitations of the HFMD-RF system should be noted. Firstly, as the system was developed in a retrospective manner that could introduce bias, a prospective validation is needed to demonstrate its predictive capability. Furthermore, we employed only one single center’s data for system development and evaluation, additional validation with local data should be carried out before its generalization to other hospitals. Due to these limitations, it would be useful to expand the model validation in prospective and multi-center clinical samples. In addition, another limitation of our study is a lack of a golden standard for severe HFMD. We, as well as other studies^15,23^, used the Chinese guidelines for HFMD diagnosis and treatment as the reference standard.

In conclusion, on a retrospective data set we successfully developed a RF model utilizing the EMR content to identify severe HFMD children in their mild stages. The novel model achieved higher sensitivity, specificity, accuracy and AUC than the existing scoring system PCIS.

## Methods

### Study population

We performed a retrospective study of hospitalizations at Guangzhou Women’s and Children’s Medical Center using data obtained from the inpatient EMR. Clinical diagnosis of HFMD was guided by the Chinese guidelines for HFMD diagnosis and treatment issued by the Ministry of Health of China (revised in 2010, http://www.moh.gov.cn/mohyzs/s3586/201004/46884.shtml). Children were clinically diagnosed as having HFMD, if they displayed maculopapular or vesicular rash on the hands, feet, oral mucosa, and/or buttock. In this study, we included 2,532 hospitalizations admitted between March 2012 and July 2015 for children <16 years suffering from newly diagnosed HFMD. Of these 2,532 children, 2,167 children were categorized as mild HFMD without any serious complications before discharge from hospital, and the other 365 children deteriorated to severe HFMD with serious complications, including encephalitis, meningitis, acute flaccid paralysis, cardiorespiratory failure or death after admission. The diagnosis of severe HFMD was reconfirmed by two senior physicians through reviewing physician progress notes.

This study was approved by the Ethics Committee and Institutional Review Board of Guangzhou Women’s and Children’s Medical Center, Guangzhou, China, and conducted in accordance with the ethical guidelines of the Declaration of Helsinki of the World Medical Association. The requirement to obtain informed consent was waived because of the retrospective nature of the study. All data were deidentified before they were provided to the investigators.

### Feature extraction

For model development, we used variables from the examination done upon admission because our aim was to identify patients who might need more attention and resources at the start of their hospitalizations. All of the variables included in the investigation were collected by reviewing the patient medical records that were preserved in EMR. In the data set, structured data elements used for severe HFMD prediction were laboratory results, which include hematologic, biochemical, immunologic, humoral and microbiological findings. Potential unstructured data elements were admission notes, which include demographic characteristics (age and sex) and clinical parameters (signs and symptoms). To extract variables from unstructured clinical documentation in Chinese, we referred to Dong Xu’s work^33^ and used a data-driven framework which incorporated machine learning and natural language processing. Specifically, the framework combined the core lexica of medical terms (Systematized Nomenclature of Medicine-Clinical Terms in Chinese, Chinese Pharmacopoeia and Wanfang Med Online, see ref.^33^), an iterative bootstrapping algorithm to obtain more accurate terms and a random forest algorithm to compute correlations between terms and descriptions. Through this procedure, the input text was converted into an output record containing a clinical variable (a symptom, a vital sign, or a demographic feature, such as papule, myoclonic jerk, convulsion, vomiting, and so on), the time of the variable and an optional description. In total, 145 variables were extracted from the structured data and 45 from the unstructured text as candidate predictors.

### Feature selection

First, we removed potentially difficult-to-obtain variables with missing rates more than 20%, leaving 153 features (126 from structured data and 27 from unstructured data) for analysis. Next, through literature review and consensus meeting between the authors including two senior physicians, nine variables (five from structured data and four from unstructured data) were selected according to importance reported by literatures. Although feature selection through using established clinical knowledge is a common method, a significant bias might be introduced in the selection process. Therefore, additional univariate analysis was used to select relevant variables for the prediction model. The top 10 variables selected by the data-driven univariate analysis based on Chi-square calculations were added to the feature set. After removal of duplicates, a total of 16 variables were selected, of which 11 were from structured data and 5 from unstructured data (Fig. 1).

### Data preprocessing

Before applying machine learning algorithms, values that were missing or out of predefined physiological ranges were interpolated with a Nearest-Neighbor algorithm^34^, which impute an incomplete variable by giving the corresponding value of the closest sample within the set of fully-informed samples. This way of interpolation is able to avoid introducing additional outliers that are nonexistent in the original dataset. The standard minimum–maximum normalization to [0, 1] was then performed for the data to reduce the effect of large feature range variation. After preprocessing, the samples in the majority groups were randomly undersampled with the EasyEnsemble method^35^ to achieve data balance.

### Model development

The random forest (RF) algorithm was adopted basing on its wide use in clinical decision systems and the excellent performance in classification tasks. A RF is a classifier that uses an ensemble of classification trees^36–39^. Each tree is unpruned (grown fully) and is built by using both bagging (bootstrap aggregation) and random variable selection, yielding low-bias and low-correlation trees. In contrast to the original publication^36^, in our models we used the Scikit-learn implementation^40^ to obtain the prediction by averaging the probability scores across the trees, rather than letting each tree vote for a single class. The number of input variables randomly chosen at each split was set as the square root of the number of features, and the number of trees in the forest was set as 500. Importance of each variable was evaluated based on the loss in predicting performance by its omission from the model.

Besides RF, a representative set of classification algorithms was also selected for the severe HFMD prediction task from the dataset. These algorithms were the support vector machine^41^, XGBoost^42^, logistic regression and multi-layer perceptrons^43^. The parameters were set to default values for the algorithms.

We developed 4 models with each algorithm for identification of patients who will progress to severe HFMD based on different numbers of variables: (1) 153 variables in the data set before feature selection; (2) 11 variables selected from structured data; (3) 5 variables from the unstructured text; (4) 16 variables from both the structured and unstructured data. All models were developed in Python using the package Scikit-learn^40^.

The holdout and cross validation methods were employed to reduce overfitting in the model and to derive a reliable estimate of the performance of the model. Mild and severe HFMD children were randomly split into two experimental datasets, a training set with 70% of cases (including 1,625 mild and 274 severe patients) and a test set with 30% of cases (including 542 mild and 91 severe patients). The training set was utilized for the feature extraction and feature selection processes described above and was used to generate the prediction models with a 10-fold cross validation step. The test set was employed to estimate the models’ performance. To measure the models’ predictive performance on practical, real world data, we calculated the sensitivity, specificity, accuracy and AUC in the training and test set^44^.

### Experimental comparison

To compare the performance of the proposed model, we also evaluated PCIS, a widely used scoring system in China. Ten physiological indexes are enrolled: heart rate, spontaneous breath rate, systolic blood pressure, oxygen partial pressure under breathing room air, serum sodium, potassium, creatinine or urea nitrogen, hemoglobin, PH value of arterial blood gas, gastrointestinal system condition (stress ulcer hemorrhage and intestinal paralysis, only stress ulcer hemorrhage or other). PCIS is usually tabulated by hand by nurses and a score of 4, 6, or 10 is generated from each category and aggregated to a 40–100 total score. Patients were divided, according to PCIS scores, into noncritical group (>80), critical group (71–80) and extremely critical group (≤70). PCIS was calculated for most of the admitted HFMD children at our medical center. For a fair comparison, we extracted the PCIS values recorded right upon admission.

### Statistical analysis

Continuous variables of each group are presented as mean ± standard deviation, and the categorical variables are expressed as absolute values. Chi-square test was used for analyzing categorical data and ratio values, while a two-sample t-test was used for analyzing the continuous data. The data that did not have a normal distribution are expressed as median (interquartile range) and were transformed logarithmically to assume a near-normal distribution for t-test. The accepted level of statistical significance for all analyses was p < 0.05. Statistical analyses were performed using SPSS version 17.0 (SPSS Inc, Chicago, IL).

## Electronic supplementary material

Two supplementary figures
