# Supplementary material for: Developing a Machine Learning System for Identification of Severe Hand, Foot, and Mouth Disease from Electronic Medical Record Data
Source: Sci Rep. 2017 Nov 27;7:16341. doi: 10.1038/s41598-017-16521-z (PMC5703994; doi:10.1038/s41598-017-16521-z)
Supplement: Supplementary file 1 — Two supplementary figures [file 41598_2017_16521_MOESM1_ESM.pdf]

Developing a Machine Learning System for Identification of Severe Hand, Foot, and Mouth Disease from Electronic Medical Record Data

Guangjian Liu[1,†], Yi Xu[2,†], Xinming Wang[3,†], Xutian Zhuang[3], Huiying Liang[1], Yun Xi[3], Fangqin Lin[1], Liyan Pan[1], Taishan Zeng[4], Huixian Li[1], Xiaojun Cao[5], Gansen Zhao[3,*], Huimin Xia[6,*]

[1]Institute of Pediatrics, Guangzhou Women and Children’s Medical Center, Guangzhou Medical University, Guangzhou, China;

[2]Department of Infectious Diseases, Guangzhou Women and Children’s Medical Center, Guangzhou Medical University, Guangzhou, China;

[3]School of Computer, South China Normal University, Guangzhou, China;

[4]School of Mathematical Sciences, South China Normal University, Guangzhou, China;

[5]Department of Research, Education and Data Management, Guangzhou Women and Children’s Medical Center, Guangzhou Medical University, Guangzhou, China;

[6]Department of Pediatric Surgery, Guangzhou Women and Children’s Medical Center, Guangzhou Medical University, Guangzhou, China.

† Guangjian Liu, Yi Xu, and Xinming Wang contributed equally to this work.

*Corresponding Authors:

Gansen Zhao, School of Computer, South China Normal University, 55 Zhongshan Road West, Guangzhou, China (gzhao@m.scnu.edu.cn);

Huimin Xia, Guangzhou Women and Children’s Medical Center, 9 Jinsui Road, Guangzhou 510623, China (xia-huimin@foxmail.com).

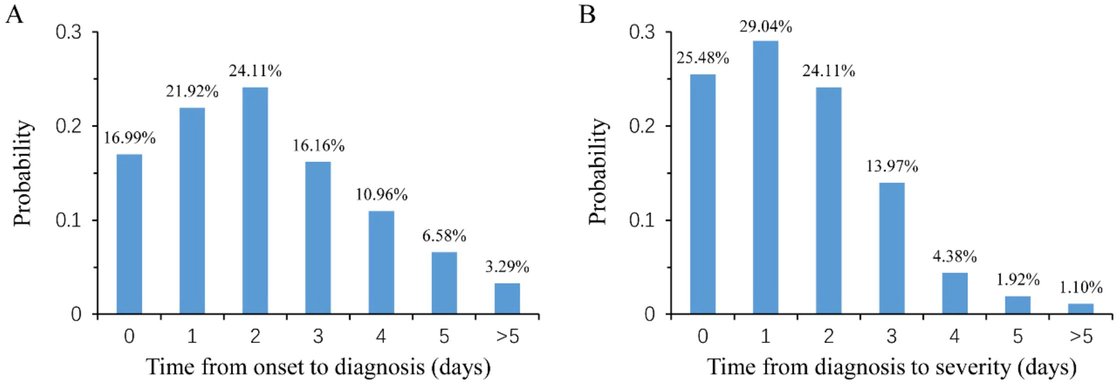

Figure S1 Distributions of onset-to-diagnosis (A) and diagnosis-to-severity (B) time interval of severe cases (n=365) of hand, foot, and mouth disease.

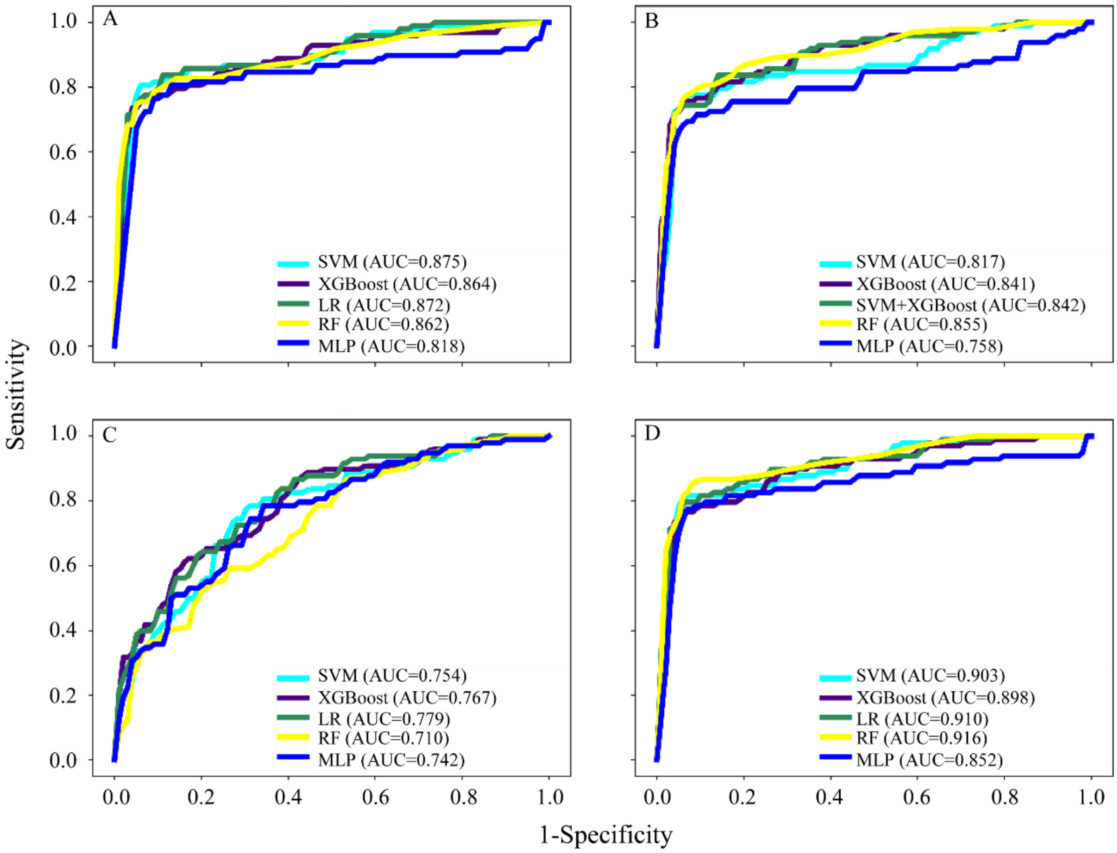

Figure S2 The receiver operating characteristic curves with five machine learning algorithms for all the 153 variables (A), 11 structured variables (B), 5 unstructured variables (C) and 16 structured and unstructured variables (D). SVM, Support Vector Machine; LR, Logistic Regression; RF, Random Forest; MLP, Multi-Layer Perceptrons.
